# Global burden of smoking-associated age-related macular degeneration: Spatiotemporal trends from 1990 to 2021 and projections to 2040

**DOI:** 10.18332/tid/205665

**Published:** 2025-07-11

**Authors:** Pengcheng Hu, Ming He, Junyang Cai, Zequn Lin, Shiying Zheng, Zihao Zhuang, Jialing Liu, Luoming Huang

**Affiliations:** 1 Department of Ophthalmology, The Second Affiliated Hospital of Fujian Medical University, Quanzhou, China; 2 Department of Ophthalmology, Dazhou Integrated Traditional Chinese and Western Medicine Hospital, Dazhou, China; 3 Department of Psychology, The School of Health, Fujian Medical University, Fuzhou, China; 4 Department of Ophthalmology and Optometry, The School of Medical Technology and Engineering, Fujian Medical University, Fuzhou, China; 5 Department of Clinical Nutrition, Chongqing University Cancer Hospital, School of Medicine, Chongqing University, Chongqing, China

**Keywords:** smoking, age-related macular degeneration, global burden of disease, sociodemographic disparities, ARIMA model

## Abstract

**INTRODUCTION:**

Smoking is a major modifiable risk factor for age-related macular degeneration (AMD); however, the long-term trends and sociodemographic disparities in its global burden remain insufficiently characterized. This study aimed to assess the evolving burden of smoking-associated AMD from 1990 to 2021 and project its trajectory to 2040.

**METHODS:**

Data from the Global Burden of Disease (GBD) 2021 database were used to extract smoking-associated AMD burden, measured by years lived with disability (YLDs) and age-standardized YLDs rate (ASYLDsR). Trends were stratified by sociodemographic index (SDI) and GBD super-regions, analyzed via estimated annual percentage change (EAPC), and projected using an autoregressive integrated moving average (ARIMA) model.

**RESULTS:**

In 2021, the global burden of smoking-associated AMD reached 58858 YLDs (a 47.1% increase from 1990), with an ASYLDsR of 2.47 per 100000 population. The burden in males significantly exceeded the female burden (45442 vs 13417 YLDs in 2021), with a widening disparity. YLDs peaked in the age group of 65–69 years (12528 cases), while ASYLDsR increased with age. East Asia had the highest cases (23248 cases, 39.5% of the global total), whereas Central Asia exhibited rising ASYLDsR. ARIMA projections estimated global YLDs to rise to 72574 (95% CI: 61319–83828) by 2040, with ASYLDsR declining to 1.54 per 100000 (95% CI: 0.90–2.17).

**CONCLUSIONS:**

The burden of smoking-associated AMD demonstrates marked demographic and geographical heterogeneity. Aging populations drive rising absolute case numbers, while disparities in tobacco control policies contribute to regional divergence in ASYLDsR.

## INTRODUCTION

Age-related macular degeneration (AMD) is the leading cause of irreversible vision loss in the elderly globally, characterized by progressive degeneration of photoreceptors and retinal pigment epithelium in the macula, ultimately leading to central vision impairment^1^. Epidemiological data estimate 196 million prevalent AMD cases worldwide, including 6.23 million moderate-to-severe vision impairments and 9% legal blindness^2,3^. AMD-related disability severely compromises quality of life and elevates risks of falls, cognitive decline, and healthcare costs.

Although the pathogenesis of AMD is not yet fully understood, environmental risk factors are widely recognized as critical contributors to disease progression^4^. Smoking, a modifiable independent risk factor, exhibits a dose-response relationship with AMD incidence, as demonstrated by multiple prospective cohort studies^5-7^. Mechanistic studies indicate that polycyclic aromatic hydrocarbons from tobacco smoke induce oxidative stress, disrupt retinal-choroidal oxygen homeostasis, accelerate drusen deposition, and promote choroidal neovascularization, thereby exacerbating macular degeneration^2^. However, systematic assessments of the long-term global burden of smoking-associated AMD and its sociodemographic determinants are lacking, hindering the development of targeted public health interventions.

The Global Burden of Disease (GBD) Study provides a standardized comparative risk assessment framework, enabling robust quantification of risk factor-attributable disease burden^8^. Prior studies using GBD data have mapped the global epidemiology of AMD, revealing significant correlations between AMD-related disability-adjusted life years (DALYs) and factors such as body mass index and urbanization rates^9^. Notably, regional analyses demonstrate lower AMD-related DALY rates in Africa and the Eastern Mediterranean compared to the Americas and Southeast Asia, with an inverse correlation to the Human Development Index (HDI)^10^, suggesting socioeconomic disparities in healthcare access or risk factor exposure modulate disease burden. However, existing research predominantly focuses on the overall epidemiology of AMD, with limited spatiotemporal analyses of smoking-associated burden or interactions between long-term trends and sociodemographic disparities.

To address this gap, we leveraged the GBD 2021 database and advanced time-series analyses to systematically evaluate the global burden of smoking-associated AMD from 1990 to 2021, projecting trends to 2040 using autoregressive integrated moving average (ARIMA) modeling. This study aims to: 1) characterize the global, regional, and sociodemographic stratification of smoking-associated AMD burden; and 2) quantify the dynamic impacts of population aging and tobacco control policies.

## METHODS

### Study design and data sources

This is a secondary dataset analysis that uses data on the burden of AMD attributable to smoking. The data used in this study were retrieved from the publicly accessible and free GBD 2021 database, which requires no special permissions. We obtained relevant data spanning from 1990 to 2021 for global, regional, and national levels, across different genders and age groups for analysis. Data were integrated from multiple heterogeneous sources, including peer-reviewed literature, census records, disease registries, electronic health records, and epidemiological surveillance reports, and standardized using the Bayesian meta-regression tool DisMod-MR 2.1^11^. This study utilizes crude numbers, years lived with disability (YLDs), reflecting the years spent with any long-term or short-term disability caused by smoking-associated AMD, and corresponding age-standardized rates to illustrate the disease burden. The GBD 2021 dataset offers global estimates of health burdens, including mortality, morbidity, and other related factors, derived from large-scale data collections analyzed using advanced modeling techniques. Therefore, the findings should be interpreted as estimates based on the available data.

### Definitions

Smokers were defined as individuals who currently engage in daily or occasional use of any tobacco smoking products, including those who have used these products in the past. This definition encompasses all forms of tobacco consumption, such as cigarettes, kretek, pipes, shisha, cigars, bidis, and other locally consumed smoking products, but excludes smokeless tobacco, e-cigarettes (also known as vaping products), and heated tobacco products. AMD was defined as a blinding disorder characterized by progressive degeneration of macular photoreceptors and retinal pigment epithelium, aligned with the GBD 2021 disease classification (ICD-10 code H35.3).

### Geographical and sociodemographic stratification

The sociodemographic index (SDI), a composite metric of per capita income, average years of education among individuals aged ≥15 years, and total fertility rate for women aged <25 years, classified countries into five tiers: low, low-middle, middle, high-middle, and high SDI. The GBD framework further partitioned 204 countries/territories into 21 super-regions and 54 sub-regions based on geographical proximity and epidemiological similarity to assess geographical heterogeneity^12^. We quantified smoking-associated AMD burden by extracting two metrics globally, across SDI-stratified regions, and for 204 countries/territories (1990–2021): 1) YLDs; and 2) age-standardized YLDs rate (ASYLDsR, per 100000 population). Data were stratified by five-year age groups (45–49 to ≥95 years) and sex.

### Statistical analysis

To analyze the dynamics of the global disease burden of smoking-associated AMD from 1990 to 2021, we investigated the corresponding age-standardized rates of YLDs. We calculated the estimated annual percentage change (EAPC) to assess trends in age-standardized rates over the study period. The EAPC was determined by fitting a linear regression model to the natural logarithm of the age-standardized rates against the calendar year, using the formula y = a + bx + e, where y denotes the natural logarithm of the age-standardized rate, x represents the calendar year, e is the error term, and b is the calculated EAPC. The sign of b indicates the direction of the trend in age-standardized rates. The 95% confidence interval (CI) for EAPC was derived using the formula 100 [exp(b) – 1]. We interpreted the trend in age-standardized rates using the EAPC and its 95% CI: an increasing disease burden is indicated if b>0 and the lower bound of the CI (LCI) >0; a decreasing disease burden is indicated if b<0 and the upper bound of the CI (UCI) <0; and the disease burden is considered stable over the period if the 95% CI includes 0^13^. To provide a clear depiction of the disease burden status, we selected data from the starting point (1990) and endpoint (2021) of the study period. These two specific years illustrate the burden at the beginning and end of the study period. We also conducted descriptive subgroup analyses to summarize patterns and trends in the burden of smoking-associated AMD across different genders, age groups, countries, and regions. No hypothesis testing or statistical inference was performed, so adjustments for multiple comparisons were not applied. We utilized the ARIMA models to forecast the prevalence of smoking-associated AMD from 2022 to 2040. This projection was presented with a 95% CI to account for potential variability in the predictions. ARIMA models are highly effective in predicting future trends based on historical data and were crucial for identifying seasonal fluctuations and underlying patterns in the time series data^14^. All analyses were conducted in R Studio (version 4.3.3), with statistical significance set at two-sided p<0.05. All key indicators have reported 95% uncertainty intervals (UI).

## RESULTS

### Global trends and gender disparities

**Figure 1:**
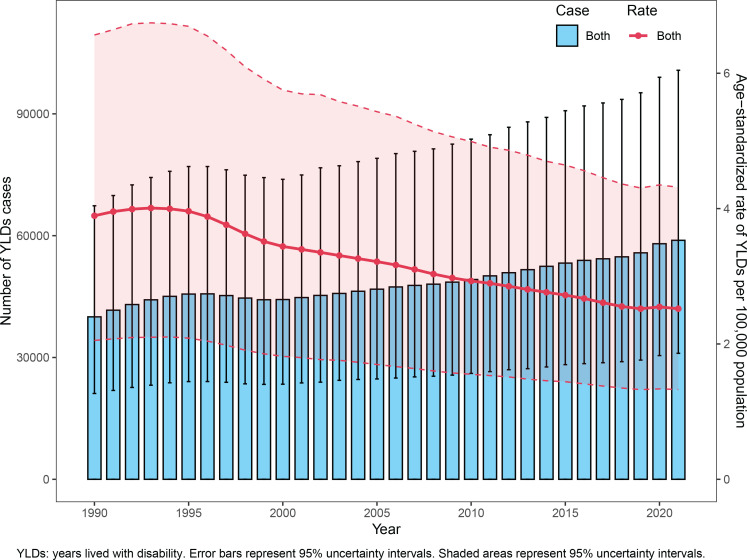
Global burden of smoking-associated AMD, 1990–2021: Trends in YLDs cases and age-standardized YLDs rate

From 1990 to 2021, the global cases of YLDs due to smoking-associated AMD significantly increased from 40006 cases (95% UI: 21136–67367) to 58858 cases (95% UI: 31031–100732), a 47.1% rise. Concurrently, the ASYLDsR declined from 3.82 per 100000 (95% UI: 2.01–6.44) to 2.47 per 100000 (95% UI: 1.3–4.23), with the EAPC of -1.66 (95% CI: -1.73 – -1.59) (Figure 1, Table 1).

**Table 1:** Age-standardized YLDs rate and temporal trends (EAPC) of smoking-associated AMD by sex, age group, SDI, and region, 1990–2021

*Characteristics*	*1990*		*2021*		*1990 to 2021*
	*Cases* *(95% UI)*	*Age-standardized* *YLDs rate per* *100000 population* *(95% UI)*	*Cases* *(95% UI)*	*Age-standardized* *YLDs rate per* *100000 population* *(95% UI)*	*EAPC* *(95% CI)*
**Global**	40006 (21136–67367)	3.82 (2.01–6.44)	58858 (31031–100732)	2.47 (1.3–4.23)	-1.66 (-1.73 – -1.59)
**Sex**					
Male	28746 (16015–46062)	1.67 (0.94–2.68)	45442 (24708–74182)	1.12 (0.62–1.84)	-1.51 (-1.58 – -1.44)
Female	11261 (5866–19049)	0.54 (0.28–0.91)	13417 (6908–22730)	0.29 (0.15–0.49)	-2.35 (-2.43 – -2.27)
**Age** (years)					
45–49	283 (119–542)	0.12 (0.05–0.23)	304 (130–592)	0.06 (0.03–0.13)	-2.35 (-2.45 – -2.24)
50–54	2068 (1023–3562)	0.97 (0.48–1.68)	2515 (1281–4384)	0.57 (0.29–0.99)	-2.04 (-2.14 – -1.94)
55–59	4989 (2637–8340)	2.69 (1.42–4.5)	6834 (3604–11522)	1.73 (0.91–2.91)	-1.63 (-1.68 – -1.58)
60–64	7901 (4233–13171)	4.92 (2.64–8.2)	10118 (5324–17019)	3.16 (1.66–5.32)	-1.63 (-1.71 – -1.55)
65–69	8333 (4506–13930)	6.74 (3.65–11.27)	12528 (6889–21214)	4.54 (2.5–7.69)	-1.51 (-1.58 – -1.44)
70–74	6586 (3552–11238)	7.78 (4.2–13.27)	10728 (5703–18761)	5.21 (2.77–9.11)	-1.52 (-1.6 – -1.43)
75–79	4865 (2552–8071)	7.9 (4.15–13.11)	7435 (3912–12751)	5.64 (2.97–9.67)	-1.4 (-1.5 – -1.3)
80–84	2892 (1454–4970)	8.17 (4.11–14.05)	4477 (2224–7697)	5.11 (2.54–8.79)	-1.84 (-1.92 – -1.75)
85–89	1465 (749–2463)	9.7 (4.95–16.3)	2567 (1301–4390)	5.61 (2.85–9.6)	-2.19 (-2.29 – -2.08)
90–94	489 (246–838)	11.42 (5.75–19.56)	1022 (506–1793)	5.71 (2.83–10.02)	-2.55 (-2.64 – -2.46)
≥95	136 (67–241)	13.33 (6.54–23.69)	332 (158–609)	6.08 (2.9–11.18)	-2.75 (-2.82 – -2.68)
**SDI region**					
High	8334 (4268–14430)	2.73 (1.39–4.73)	8774 (4373–15504)	1.49 (0.75–2.63)	-2.09 (-2.2 – -1.98)
High-middle	11014 (5769–18458)	4.14 (2.15–6.95)	17829 (9451–30413)	3.2 (1.69–5.46)	-1.03 (-1.14 – -0.93)
Middle	10857 (5820–18257)	4.07 (2.18–6.83)	19636 (10296–33391)	2.66 (1.4–4.53)	-1.82 (-1.96 – -1.68)
Low-middle	7823 (4146–13068)	4.98 (2.64–8.3)	9767 (5094–16697)	2.58 (1.35–4.41)	-2.29 (-2.42 – -2.17)
Low	1946 (1003–3230)	3.41 (1.76–5.65)	2816 (1436–4858)	2.21 (1.13–3.82)	-1.61 (-1.7 – -1.52)
**Region**					
Andean Latin America	81 (38–153)	1.53 (0.71–2.88)	202 (92–381)	1.27 (0.58–2.41)	-0.73 (-0.8 – -0.67)
Australasia	121 (55–231)	1.86 (0.83–3.57)	141 (59–278)	0.94 (0.4–1.85)	-2.1 (-2.18 – -2.02)
Caribbean	67 (31–125)	0.96 (0.44–1.79)	88 (41–164)	0.59 (0.27–1.1)	-1.62 (-1.71 – -1.52)
Central Asia	139 (70–247)	1.07 (0.54–1.89)	234 (11–422)	1.04 (0.52–1.86)	0.13 (-0.03 – 0.28)
Central Europe	1032 (539–1780)	2.44 (1.27–4.22)	1039 (536–1819)	1.72 (0.89–3.02)	-1.21 (-1.25 – -1.16)
Central Latin America	363 (184–640)	1.71 (0.87–3.01)	485 (241–877)	0.71 (0.35–1.29)	-2.92 (-3.01 – -2.83)
Central Sub-Saharan Africa	15 (7–29)	0.27 (0.13–0.51)	32 (14–60)	0.23 (0.1–0.42)	-0.32 (-0.51 – -0.13)
East Asia	9970 (5358–16669)	4.43 (2.38–7.41)	23248 (12381–39444)	3.73 (1.98–6.33)	-1.15 (-1.4 – -0.91)
Eastern Europe	546 (257–970)	0.69 (0.32–1.22)	599 (291–1051)	0.61 (0.3–1.07)	-0.42 (-0.73 – -0.11)
Eastern Sub-Saharan Africa	582 (298–992)	3.12 (1.6–5.31)	690 (339–1218)	1.61 (0.79–2.83)	-1.93 (-2.12 – -1.74)
High-income Asia Pacific	729 (352–1277)	1.35 (0.65–2.36)	875 (415–1604)	0.67 (0.32–1.24)	-2.4 (-2.48 – -2.32)
High-income North America	1505 (741–2691)	1.53 (0.75–2.73)	1715 (777–3209)	0.91 (0.41–1.69)	-1.83 (-2.04 – -1.63)
North Africa and Middle East	2932 (1505–4985)	6.62 (3.4–11.29)	5614 (2894–9738)	4.71 (2.42–8.17)	-1.16 (-1.19 – -1.12)
Oceania	11 (5–21)	1.34 (0.61–2.48)	19 (9–37)	0.91 (0.42–1.74)	-1.07 (-1.15 – -0.98)
South Asia	8928 (4715–14918)	6.11 (3.23–10.17)	10540 (5435–18280)	2.71 (1.4–4.7)	-2.95 (-3.12 – -2.78)
South-East Asia	2600 (1368–4404)	4.05 (2.13–6.85)	3843 (1971–6547)	2.18 (1.12–3.7)	-2.25 (-2.35 – -2.14)
Southern Latin America	168 (74–323)	1.32 (0.57–2.54)	187 (81–365)	0.79 (0.34–1.53)	-1.61 (-1.71 – -1.52)
Southern Sub-Saharan Africa	96 (48–170)	1.4 (0.69–2.47)	107 (52–196)	0.7 (0.34–1.27)	-2.12 (-2.19 – -2.05)
Tropical Latin America	668 (342–1179)	2.82 (1.44–4.97)	1072 (514–1968)	1.51 (0.72–2.79)	-2.01 (-2.28 – -1.74)
Western Europe	9058 (4615–15579)	5.65 (2.88–9.72)	7408 (3686–13083)	2.88 (1.44–5.07)	-2.21 (-2.28 – -2.14)
Western Sub-Saharan Africa	394 (198–706)	1.7 (0.86–3.05)	722 (356–1300)	1.38 (0.68–2.49)	-1.05 (-1.22 – -0.87)

AMD: age-related macular degeneration. YLDs: years lived with disability. UI: uncertainty intervals. CI: confidence interval. SDI: socio-demographic index. EAPC: estimated annual percentage change.

Male YLDs substantially exceeded female YLDs [2021: 45442 cases (95% UI: 24708–74182) vs 13417 cases (95% UI: 6908–22730)]. Females exhibited a greater decline in ASYLDsR (EAPC= -2.35 vs males: -1.51), yet the absolute disparity widened (Supplementary file Figure 1). Specifically, male ASYLDsR decreased from 1.67 per 100000 (95% UI: 0.94–2.68) in 1990 to 1.12 per 100000 (95% UI: 0.62–1.84) in 2021, while female ASYLDsR declined from 0.54 per 100000 (95% UI: 0.28–0.91) to 0.29 per 100000 (95% UI: 0.15–0.49) (Table 1).

### Age-stratified and regional variations

**Figure 2:**
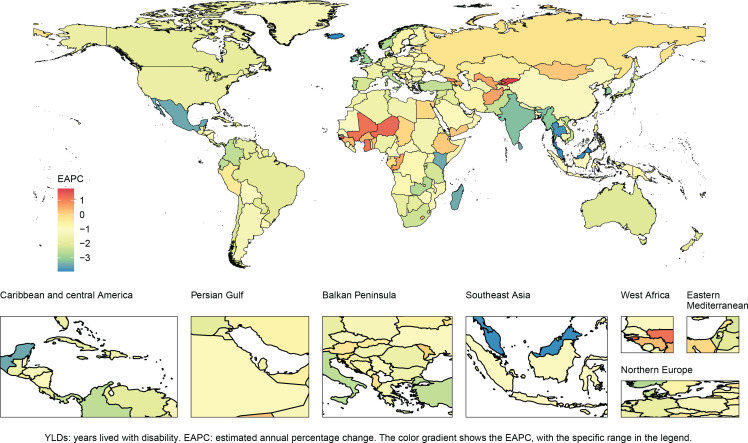
Heterogeneity in smoking-associated AMD burden across 204 countries and territories: Estimated annual percentage change (EAPC) in age-standardized YLDs rate, 1990–2021

YLDs exhibited an inverted U-shaped distribution by age, peaking in the age group of 65–69 years (12528 cases) before declining. ASYLDsR increased steadily with age (Supplementary file Figure 2). Regionally, East Asia had the highest YLDs [2021: 23248 cases (95% UI: 12381–39444), 39.3% of global burden], while Oceania had the lowest (19 cases; 95% UI: 9–37). Central Asia showed rising ASYLDsR [EAPC=0.13 (95% CI: -0.03 – -0.28)], whereas South Asia had the largest decline [EAPC= -2.95 (95% CI: -3.12 – -2.78)] (Table 1, Figure 2).

Stratified by SDI, the high-middle SDI regions had the highest ASYLDsR (3.2 per 100000 in 2021), while high SDI regions had the lowest ASYLDsR (1.5 per 100000) and the steepest decline (EAPC= -2.09). Females in low-middle SDI regions experienced the largest ASYLDsR reduction [EAPC= -2.82 (95% CI: -3.03 – -2.61)] (Supplementary file Figure 3).

### Country-specific burden and projections

China reported the highest smoking-associated AMD cases [2021: 23157 cases (95% UI: 12331–39278)] but a slow decline in ASYLDsR [EAPC= -1.17 (95% CI: -1.41 – -0.92)]. Iceland achieved the largest ASYLDsR reduction [EAPC= -3.96 (95% CI: -4.20 – -3.71)], while Kyrgyzstan showed a significant increase [EAPC=1.81 (95% CI: 1.55–2.07)] (Table 1, Figure 2).

ARIMA projections indicated that global YLDs will rise to 72574 cases (95% CI: 61319–83828) by 2040, with ASYLDsR decreasing to 1.54 per 100000 (95% CI: 0.90–2.17). Males will experience a greater burden increase (2022–2040: +22.9% vs females: +2.1%) (Supplementary file Figure 4).

## DISCUSSION

Leveraging GBD 2021 data, this study systematically assessed the evolving burden of smoking-associated AMD from 1990 to 2021 and projected trends to 2040 via ARIMA modeling. Results revealed a persistent increase in smoking-associated AMD cases alongside a significant decline in the ASYLDsR. This paradox may reflect global advancements in diagnostics and therapies. Clinical trials demonstrated the efficacy of intravitreal anti-vascular endothelial growth factor (VEGF) antibodies, treated patients have shown substantial visual improvement and reduced disease burden^15^. Additionally, marked gender, age, and regional disparities in smoking-associated AMD cases highlight the dual challenges of population aging and growth.

The cases of global YLDs for smoking-associated AMD rose by 47.1% (1990–2021), while ASYLDsR declined, a paradox driven by accelerated population aging. The peak YLDs in the age group of 65–69 years (12528 cases in 2021) align with AMD’s age-dependent pathology, reflecting cumulative smoking exposure in older adults. The Gutenberg Health Study (GHS) indicates that AMD incidence and AMD progression were associated with higher age; for each 10-year increase in age, the risk of AMD doubles (RR=2.30)^16^, reinforcing aging as a key driver. Although tobacco control reduced age-specific incidence (ASYLDsR decline), expanding elderly populations increased absolute case numbers – a trend mirrored in neurodegenerative diseases like Alzheimer’s^17^. Therefore, routine retinal screening and early interventions for elderly smokers are critical to mitigating AMD-related vision loss.

Gender analysis revealed significantly higher YLDs and ASYLDsR in males, with widening absolute disparities – contrary to the female-predominant burden in non-smoking-associated AMD. Previous studies showed higher AMD prevalence and burden in females^18^, highlighting smoking’s sex-specific risk. Globally, male smoking rates persistently exceed female rates^19^. In China, approximately 300 million smokers consume 40% of global cigarettes, with males comprising 96% of smokers^20^, likely driving the expanding gender gap. Epidemiological studies underscore a strong smoking-AMD association. A study on East Asian males reported a 50% higher risk of neovascular AMD in smokers versus never-smokers^21^. A 12-year prospective cohort of 61862 women found that those smoking ≥25 cigarettes/day had over twice the AMD risk compared to non-smokers^6^. Similarly, a 7-year cohort of 21157 men showed AMD incidence of about 2% in those smoking ≥20 cigarettes/day versus <1% in never smokers^5^. Former smokers also faced elevated risk (odds ratio: 1.7 vs never smokers)^22^. Smoking reduces choroidal capillary blood flow, inducing ischemia and microinfarction^23^, while hemodynamic changes exacerbate retinal hypoxia and promote neovascularization, increasing neovascular AMD risk^24^. Males face prolonged smoking exposure and lower cessation rates^25^, with sex-specific tobacco metabolism amplifying oxidative stress and choroidal damage^24^. However, smoking cessation markedly reduces risk; former smokers’ AMD risk approaches never smokers’ levels after 20 years of abstinence^26^. These findings underscore cessation as a critical preventive strategy. Despite steeper ASYLDsR declines in females, persistent male burden necessitates tailored interventions for middle-aged/older males.

SDI-based analysis revealed significant disparities in smoking-associated AMD burden across regions of varying development levels. High-middle SDI regions exhibited the highest ASYLDsR (2021), while high SDI regions had the lowest ASYLDsR with the steepest decline. This contrast likely reflects divergent tobacco control policies: high SDI regions typically possess advanced diagnostics and therapies, coupled with health education campaigns enhancing public awareness of AMD and preventive measures. Early screening and timely interventions in these regions mitigate severe outcomes like blindness, thereby reducing disease burden. Concurrently, high SDI nations reduced smoking prevalence through stringent legislation and public education, whereas high-middle SDI regions lagged in tobacco control despite rapid economic growth, failing to address aging-driven demographic shifts^27^. South Asia achieved the largest ASYLDsR reduction, attributable to National Tobacco Control Program (NTCP) implementation^28^, while Central Asia’s rising burden may stem from traditional smoking practices (e.g. waterpipe) and insufficient health outreach^29^.

China bears the highest global burden of smoking-associated AMD, linked to its unique smoking patterns. As the world’s largest tobacco producer and consumer, China has about 300 million smokers^20^. In 2019–2020, smoking prevalence among adults ≥40 years was 27.2%, with males (52.1%) smoking 21 times more than females, and daily consumption (18 cigarettes) exceeding global averages^30^. Projections suggest 2 million annual tobacco-related deaths by the 2030s, rising to 3 million by 2050^31^. Despite governmental cessation clinics and a national quitline, services remain underutilized. By 2018, the overall quit rate was about 20%, with only 3–5% of smokers using quitlines, medications, or clinics^32^. Compared to WHO MPOWER policies, China’s tobacco control lags in: 1) national smoke-free legislation; 2) taxation (current rates below WHO’s 75% threshold); and 3) health communication (absent graphic warnings on packaging)^33^. These gaps reflect synergies between China’s vast smoking population and weak policy enforcement. These findings emphasize the need for localized WHO FCTC implementation in low-income countries.

ARIMA projections indicate rising global YLDs (+22.9% in males vs +2.1% in females by 2040) despite declining ASYLDsR, highlighting aging’s inexorable impact. Male-predominant burden growth necessitates dual strategies: 1) reducing smoking via legislation (e.g. plain packaging, e-cigarette regulation)^34^; and 2) enhancing retinal screening and early interventions (e.g. anti-VEGF therapy) for high-risk groups (male smokers aged ≥65 years )^35^.

### Limitations

This study has several limitations. First, the EAPC analysis assumes a linear trend, which may not fully capture complex temporal changes. Although ARIMA models are effective for time-series data, all forecasting models have inherent uncertainty, and actual outcomes may differ due to unpredictable social, economic, policy, and environmental factors. Second, while the study adjusts for age and known confounders using standardization, unmeasured or inadequately adjusted confounders like genetic susceptibility and diet may still influence risk attribution. Additionally, in some cultures, the stigma around female smoking may lead to reporting bias. The study primarily focuses on smoking’s impact on AMD burden and does not fully consider the emerging treatments for AMD, such as the adoption and efficacy of anti-VEGF therapies. Despite the GBD study’s efforts to integrate multi-source data, data accuracy and completeness may be low in regions with limited reporting, especially in low-income areas, where the 95% UI is wider, reflecting lower precision of these estimates. This could be related to data sparsity or quality issues in these regions. Furthermore, model parameter selection is crucial for prediction accuracy. Although the best parameter combinations were chosen based on the AIC criterion, different choices could lead to different results. Lastly, SDI and regional classifications may mask subnational heterogeneity in smoking rates and healthcare access.

## CONCLUSIONS

Smoking-associated AMD exhibits marked demographic and geographical heterogeneity. This study provides information to optimize global tobacco control strategies, emphasizing the integration of retinal health into aging-focused policies.

## Data Availability

The data are accessible and can be downloaded through the official website of the Institute for Health Metrics and Evaluation (IHME) (http://ghdx.healthdata.org). Given the open-access nature of this database and the absence of personally identifiable information, our study adheres to the ethical standards for the utilization of public data.
